# The New Year

**Published:** 1890-01

**Authors:** 


### HALL'S JOURNAL OF HEALTH

TRUTH DEMANDS NO SACRIFICE; ERROR CAN MAKE NONE.

VOL. 37. JANUARY, 1890. NO. 1.

### THE NEW YEAR.

We extend to all the patrons of our JOURNAL, in whatever relation, and to all former patrons who have withdrawn their support for any cause, a most cordial greeting of the New Year, and our best wishes for their individual prosperity, happiness and long life.

It has always been, as it always will be, our endeavor to place before our readers such simple guides to health as one can make avail of without special training, or giving up ordinary avocations. It has also been our endeavor to free the mind of certain distracting bugbears, which not only conduce to disease, but oftentimes stand grimly in the way of recovery. For this we have not passed without censure on the part of a few who seem to be so tenaciously wedded to old and decaying theories, founded in misconception and nursed by bigotry, as to preclude the entrance of any new light. There is always an ultra-conservative class in every community of a dozen or more members, which adh ring to the past, timerously reject the advancement of new ideas. It is the same order that opposed the building of turnpikes and the introduction of power looms in the mother country, as contrary to the established order of things, and destructive of the means of livelihood of hand-workmen and small inn-keepers who thrived on mud roads and snail-pace hand spinning and weaving.

The rule of the century, now in its last decade, is universal progress. Advances in art, in science, in mechanics, and more than all, in discovery and invention, are the order upon every hand, and forward still is the resistless march of improvement. The range of genius is from the unexplored recesses beneath the earth's surface to the vast region of worlds by which it is encircled, making subservient to the human will the more powerful and mysterious forces of nature. Is it to be wondered at then, that amid these things which have wrought such changes in means and methods, theories and ideas more or less speculative and problematical should change and readjust themselves to altered conditions and newly discovered truths; that even the foundations of creeds and philosophies should be re-examined and readjusted in the light of the present? Only a comparatively short time ago, the Roman hierarchy and Calvinistic intolerance alike, burned to the stake noble men and women who opposed their dogmas with truths which they have since been forced to accept from their unanswerable logic and demonstration. The grand Lama of Thibet still inculcates as an essential element of religious belief, that the world is a vast plane, resting upon a fixed foundation, and listens with incredulity to the tales of navigators who assert its spheroidity and axial motion.

Slowly and stubbornly enough the untenable fields of error are being abandoned for firmer ground, but the grim old orders leave them under pressure, like the confused columns of a retreating army, with its rear covered by cannon and blazened with fire.

We shall continue to present such leading truths as serve to free the mind of ill-founded apprehension and conduce to its requisite tranquility and healthfulness. That the great majority of our readers appreciate our efforts as sanitary helps is shown by our largely increased circulation, and it is solely in their interest, as by their patronage, that our publication is upheld.

